# IGF1R inhibition and PD-1 blockade improve anti-tumor immune response in epithelial ovarian cancer

**DOI:** 10.3389/fonc.2024.1410447

**Published:** 2024-10-10

**Authors:** Lina Somri-Gannam, Shilhav Meisel-Sharon, Shay Hantisteanu, Tomer Bar-Noy, Emiliya Sigal, Gabriel Groisman, Mordechai Hallak, Haim Werner, Ilan Bruchim

**Affiliations:** ^1^ Gynecology Oncology Laboratory, Department of Obstetrics and Gynecology, Hillel Yaffe Medical Center, Hadera, Israel; ^2^ The Ruth and Bruce Rappaport Faculty of Medicine, Technion - Israel Institute of Technology, Haifa, Israel; ^3^ Gynecology and Gynecologic Oncology Department, Hillel Yaffe Medical Center, Hadera, Israel; ^4^ Institute of Pathology, Hillel Yaffe Medical Center, Hadera, Israel; ^5^ Department of Human Molecular Genetics and Biochemistry, Sackler School of Medicine, Tel Aviv University, Tel Aviv, Israel

**Keywords:** insulin-like growth factor-1 receptor, epithelial ovarian cancer, immunotherapy, dendritic cells, PD-1, immune system

## Abstract

**Introduction:**

The insulin-like growth factor (IGF) system plays a key role in regulating growth and invasiveness in epithelial ovarian cancer (EOC) and is considered a promising therapeutic target. EOC is an immunosuppressive disease, although there are limited data about the involvement of the IGF1R system in the anti-tumor immune response in the EOC microenvironment.

**Methods:**

In the current study, we hypothesized that IGF 1 receptor (IGF1R) involvement in the maturation of dendritic cells (DC) with the co-inhibition of IGF1R and PD-1 would affect the EOC microenvironment.

**Results:**

We found that DC pretreated with IGF1R inhibitor resulted in fewer EOC cells. Moreover, *in vivo* experiments conducted with an EOC mouse model, with anti-PD-1/IGF1R combined, resulted in lower tumor weight compared to individual treatments. Additionally, anti-PD-1/IGF1R treatment increased DC by 34% compared with AEW-541 and 40% with anti-PD-1. The combined treatment increased CD8+ T-cell levels compared to AEW-541 alone. RNA-seq data analysis indicated that anti-PD-1/IGF1R led to a more potent immune response, as reflected by altered gene expression levels related to anti-tumor immune response, compared with either treatment alone.

**Discussion:**

These findings provide novel evidence that IGF1R axis inhibition combined with PD-1 blockade may be an effective therapeutic strategy for selected EOC patient populations.

## 1 Introduction

Epithelial ovarian cancer (EOC) remains the most lethal gynecological malignancy worldwide, accounting for 90% of all ovarian tumors (1). Due to the late onset of symptoms, over 80% of patients are diagnosed at advanced stages, and only 45% survive five years after diagnosis (2). Therefore, novel treatments are required to improve patient survival. As EOC is an immunosuppressive disease (3), many factors in its microenvironment can interfere with the presence or activity of tumor-infiltrating leukocytes (TIL); thus, enabling cancer progression. Hence, various immunotherapeutic strategies aiming at shifting the balance from immunosuppression to immune surveillance have been developed (4–7). Immunotherapy has recently emerged as a promising complementary approach to standard ovarian cancer (OC) treatments (4).

The programmed cell death protein-1 (PD-1) and its ligand (PD-L1) are immune checkpoints that, when targeted, can reverse tumor-mediated immunosuppression (8). PD-1 is expressed in activated T and B cells, natural killer (NK) cells and dendritic cells (DC), and delivers inhibitory signals in lymphocytes by interacting with its ligand PD-L1 (B7-H1), which is expressed in tumor cells (9). This interaction leads to T cell death (10) and to the suppression of cytokine secretion, such as IFN-α, TNF- γ, and IL-2, by inhibiting PI3K activity (8). Studies have shown that the PD1/PD-L1 pathway is associated with poor prognosis in women with OC (11–13). However, the clinical response of patients with EOC to treatment with the immune checkpoint inhibitors PD-1 or PD-L1 has been modest in comparison with the robust response observed in melanoma, lung cancer, and renal cell cancer (5, 14). Ovarian tumors contain an abundance of immune cells, including lymphocytes and DC (8, 15). DC directly enhance pathogenesis through the release of various cytokines and chemokines, which together form an integrated pathologic network (16–18). Thus, optimal DC function is necessary for the initiation and maintenance of protective anti-tumor immunity. OC potently suppresses the anti-tumor immune response by provoking DC dysfunction in the tumor microenvironment (TME). Specifically, the TME modifies DC function by inhibiting DC activation or maturation, while inducing immunosuppressive DC and other related myeloid cell populations (19). Given that, DC immunotherapy can increase the number of efficient mature DC and consequently, generate anti-tumor specific T cells (20).

Preclinical and early clinical data have confirmed the ability of DC vaccines to induce potent immune responses that in some instances can lead to measurable clinical responses (21, 22). A phase I/II study demonstrated that autologous DC vaccines are a safe and feasible therapy for advanced ovarian and primary peritoneal cancers in remission (23). The study included 11 patients, and the autologous DC vaccine elicited a modest immune response by presenting tumor antigens and improved overall 3-year survival in 54% and 2-year survival in 9% of patients (23). In addition, a recent phase I study demonstrated that DC vaccination followed by adaptive cell therapy were feasible and produced antitumor immunity and clinical benefit without adverse events (24). Moreover, Tanyi et al. showed that DC vaccination in platinum-treated patients and those with recurrent OC, led to significantly increased survival and improvement in median progression-free survival from 4.1 to 11.1 months (25). These new insights become particularly important in the context of our study and in discovering a potential new therapy for patients with EOC.

Interestingly, a study aiming to increase DC efficiency by applying immunotherapeutics showed that the insulin-like growth factor-1(IGF1) axis affects DC maturation and T cell activation (26). The IGF family consists of growth factors, binding proteins and receptors that play a key role in regulating growth, survival and cell differentiation (27). Interaction of the IGF1R with its ligand leads to the activation of several intracellular secondary messenger pathways, including the Ras-Raf-MAPK and PI3K/AKT signaling cascades (28). IGF1R overexpression has been linked to the development of several cancers, including ovarian (29–37). *In-vitro* studies show that inhibition of the IGF1 signaling pathway suppresses OC cell survival (30); however, results from clinical trials targeting the IGF1R have been disappointing. In addition to the expression of IGF1R in cancer cells, IGF1R is expressed in immune cells (31–34); although, the exact function of IGFs on host immunity and immune cells such as DC remains unclear. This study examined the involvement of the IGF1R pathway in DC differentiation in EOC and evaluated the effects of IGF1R targeting combined with anti-PD1 on EOC proliferation and TME in an OC mouse model.

## 2 Materials and methods

### 2.1 Mice and cell lines

The human OC cell lines ES2 and SKOV3 were provided by Prof. Ilan Tsarfaty (Tel Aviv University). Cells were maintained in DMEM with 10% FBS, 2 mM glutamine and 100 μg/ml streptomycin in the presence of 5% CO_2_. The Kuramochi (Ku) OC cell line, obtained from Dr. Ruth Perets (Rambam Health Care Campus, Technion), was grown in RPMI media supplemented with 10% FBS. THP-1, a human monocyte cell line derived from a patient with acute monocytic leukemia, was obtained from Prof. Isaac Witz (Tel Aviv University) and cultured in RPMI-1640 medium. Media were supplemented with 10% FBS, 2 mM glutamine, and 100 μg/ml streptomycin in the presence of 5% CO_2_. In addition, 10 mM HEPES, 1 mM sodium pyruvate, 1500 mg/L sodium bicarbonate and 0.05 mM 2-mercaptoethanol were added. A mouse ovarian epithelial papillary serous adenocarcinoma cell line, ID8, was donated by Dr. Katherine F. Roby (38) (University of Kansas Medical Center, KS, USA). All reagents were purchased from Biological Industries, Kibbutz Beit Haemek, Israel. Female C57BL/6 (B6) mice were purchased from ENVIGO RMS Co. (Jerusalem, Israel) and housed under pathogen-free conditions at the Technion – Israel Institute of Technology.

### 2.2 Reagents

The NVP-AEW541 (AEW) selective IGF1R inhibitor was obtained from MedChemExpress MCE (Suite Q NJ, USA). For *in-vitro* use, the AEW was kept as a stock solution (10 mM) in DMSO and for *in-vivo* use, it was dissolved in 0.2 mL of 25 mmol/L L(+)-tartaric acid. A PD-1 inhibitor was purchased from BioXCell (NH, USA, Cat. BE0146). In some of the experiments, cells were treated with IGF1 (50 ng/ml) (Peprotech, Rehovot, Israel). Mice antibodies for flow cytometry were obtained from Rhenium: CD3 (CAT. 56-0033-82), CD4 (CAT. 48-0041-82), CD8 (CAT. 69-0081-82); CD45 (CAT. 25-0451-82); CD11b (CAT. 53-0112-82); CD11c (CAT. 47-0114-82), CD86 (CAT. 12-0861-82) and Fc Receptor Blocking Solution (B223505) from Biolegend (San Diego, CA, USA). Human antibodies for flow cytometry were obtained from Biolegend: CD45 (CAT. 368530), CD11c (CAT. 371506), CD1c (CAT. 331520), Zombie Violet™ Fixable Viability Kit (CAT. 423114) and carboxyfluorescein succinimidyl ester (CFSE) (CAT. 423801).

### 2.3 Induction of differentiation of immature DC

THP-1 cells were harvested by centrifugation, resuspended in complete RPMI medium at a concentration of 2×10^5^ cells/ml, and transferred in a final volume of 18 ml into six-well culture plates. To induce differentiation, 20 ng/ml IL-4 and 20 ng/ml GM-CSF were added. Cells were cultured for 5 days to acquire the properties of immature DC. Medium was exchanged every 2 days with fresh cytokine-supplemented medium in a humidified incubator at 37°C and 5% CO_2_.

### 2.4 Induction of differentiation of mature DC

Mature DC were generated from immature DC (5-day culture) by adding 20 ng/ml TNF-α and 200 ng/ml ionomycin in serum-free culture medium at a concentration of 2×10^5^ cells/ml for 2 days (7-day culture based on the “5 + 2” protocol (39), in a humidified incubator at 37°C and 5% CO_2_.

### 2.5 Proliferation assays

For proliferation assays, 4 x 10^6^ cells/mL of SKOV3, ES2 and Ku were incubated with CFSE working solution for 20 minutes at room temperature (RT) in the dark. Staining was quenched by adding 5 times the original staining volume of cell culture medium containing 10% FBS. Next, SKOV3, ES2 and Ku-stained cells were co-cultured with AEW-treated-DC on six-well culture plates, for 48 h. After this period, cells were harvested and run on a Navios EX flow cytometer (Beckman Coulter, Brea CA, USA) and analyzed using Kaluza Software. Each experiment was repeated at least three times, and a representative experiment is presented.

### 2.6 ID8-lentiviral transduction

A lentiviral vector equipped with mCherry fluorescent protein and G418 resistance was obtained from Prof. Ilan Tsarfaty (Tel Aviv University). A 1*10^5^/ml ID8 cell line was incubated with the mCherry-Lentiviral vectors and 8 µg/ml polybrene on 12-well culture plate, for 48–72 hours. The cells were then split into replicate plates, one subjected to G418 antibiotic (600 µg/ml) selection, while the other was maintained in non-selective media. Following antibiotic selection, the replicate cultures were analyzed using a Nikon ECLIPSE Ti microscope and IVIS 200 imaging system.

### 2.7 Animal studies

Thirty-two mice were injected intraperitoneally (IP) with 3.5 × 10^6^ ID8-mCherry cells in 0.1 mL of PBS. Fourteen days post-injection, mice were weighed and randomized into 4 treatment groups (8 mice/group): Control, PD-1 inhibitor, IGF1R inhibitor, and PD-1 and IGF1R inhibitors combined. The PD-1 inhibitor was administered IP at a dose of 200 μg twice per week for 2 weeks. IGF1R inhibitor (AEW) treatment was administered by oral gavage, at a dose of 50 mg/kg, twice daily for 7 days. Mice from the control group were injected with antibody dilution buffer. The weight and abdominal girth of the mice were measured every 3 to 4 days. Tumor progression was checked via *in-vivo* animal imaging (ultrasound and IVIS 200 imaging system). The mice were checked daily for clinical signs of a swollen belly, indicative of ascites and for evidence of toxicity, such as changes in behavior, mobility, respiratory distress, weight loss, diarrhea, hunched posture and failure to eat or drink. Following institutional guidelines, the mice were euthanized when they developed ascites, had a weight increase over 30% of their original day 1 weight or if they showed any evidence of toxicity. Survival of each mouse was recorded, and overall survival (OS) was calculated. At the end of the study, the remaining mice were euthanized, and tumors were harvested; tumor weights were measured in each group.

### 2.8 Flow cytometry assays

#### 2.8.1 *In-vitro* experiments

For cell surface staining, THP-1 and differentiated DC were incubated with Zombie Violet™ Fixable Viability Kit in the dark for 15-30 min at RT, to check the live/dead cells. Then, the cells were incubated with human antibodies against CD141, CD1c, CD11c, CD11b for 20 min on ice in the dark. Staining was terminated by adding fluorescence activated cell sorting (FACS) buffer (PBS with 3% FBS and 2 mM EDTA). For intracellular staining, THP-1 cells were washed twice with PBS and fixed by using 4% paraformaldehyde for 15 min at RT. Cells were then washed with PBS and permeabilized by adding 1% Triton for 10 min at RT. Cells were then washed and incubated with primary antibodies against total IGF1R (sc-81,167, Santa Cruz Biotechnology, Inc.) and phospho IGF1R (Y1135/1136, Cell Signaling) for 1 h at RT. Cells were then washed and incubated with secondary antibodies Alexa flour donkey anti-mouse (715-545-150, Jackson Immuno Research, West Grove, PA, USA) and CY3 donkey anti-rabbit antibodies (7111-165-152, Jackson Immuno Research) for 20 min in the dark, on ice. Staining was terminated by adding FACS buffer. Each experiment was repeated at least three times, and a representative experiment is presented.

#### 2.8.2 *In-vivo* experiments

Flow cytometry was used to analyze the tumor immune microenvironment in the mouse model. Tumor tissues were dissected, weighed, minced and incubated in RPMI 1640 medium containing 2% FBS, 1 mg/ml collagenase IV (Sigma: #C5138), and 200 U DNase I (Sigma: H6254) at 37°C for 1 h to obtain a cell suspension. Cells from tumor tissues were blocked with anti-mouse Fc Receptor Blocking Solution (1:1000) and then stained with mouse antibodies against CD45, CD11b, CD11c, CD3e, CD8a and CD4. Samples were run on a Navios EX flow cytometer and analyzed using Kaluza Software. The lymphocyte and DC populations were selected by gating CD45 positive cells.

### 2.9 RNA-seq assay

RNA-seq analyses were conducted on 13 frozen tumor samples divided into 4 biological groups: Control – untreated mice (group 1, n=2); IGF1R inhibitor-treated mice (group 2, n=4); PD1 inhibitor-treated mice (group 3, n=3); and combination-treated mice (group 4, n=4). RNA-seq analyses were done at the Technion Genomics Center (Haifa, Israel).

#### 2.9.1 RNA extraction and quality control

Frozen tumor tissue samples were disrupted in 600 µl buffer RLT and homogenized by Kinematica AG homogenizer. The lysate was then centrifuged for 3 min at maximum speed and the supernatant was loaded on a Qiacube (Qiagen) for automated RNA extraction with RNeasy kit (cat no. 74106). The quality of the RNA was evaluated using the TapeStation 4200 (Agilent) with the RNA kit (cat no. 5067-5576). The RIN values of all samples were in the range of 6.8-9.3, indicating good quality.

#### 2.9.2 Library preparation

Thirteen RNA-seq libraries were constructed simultaneously according to the manufacturer’s protocol (NEBNext Ultra II Directional RNA Library Prep Kit for Illumina, cat no. E7760) using 800 ng total RNA as starting material. mRNA pull-down was performed using the Magnetic Isolation Module (NEB, cat no. E7490). After construction, the concentration of each library was measured using Qubit (Invitrogen) and the size was determined using the TapeStation 4200 with the High Sensitivity D1000 kit (cat no. 5067-5584). All libraries were mixed into a single tube with equal molarity. The RNA-seq data was generated on Illumina NextSeq2000, using P2 100 cycles (Read1-100; Index1-8; Index2-8) (Illumina, cat no. 20046811) (**Table 1**).

**Table 1:** Sample statistics summary.

Sample ID	Number of reads	Number uniquely mapped reads	% Uniquely mapped reads	% Multi- mapped reads	% Unmapped reads
1_1_Both	41,430,829	36,637,000	88.43	9.15	0.65
1_2_Both	40,107,491	35,658,639	88.91	8.93	0.62
2_1_1L	40,338,627	35,352,451	87.64	10.4	0.79
2_1_1R	43,294,942	38,675,833	89.33	9.15	0.63
2_1_Non	42,834,864	38,287,788	89.38	9.17	0.63
2_2_Non	42,578,501	37,993,687	89.23	9.17	0.62
3_1_1L	39,070,755	34,129,161	87.35	10.36	0.67
3_2_1L	40,722,656	35,927,741	88.23	9.65	0.64
3_2_1R	42,292,843	36,934,737	87.33	10.84	0.71
4_1_1L	40,240,086	35,599,660	88.47	9.91	0.76
4_1_Both	42,948,345	37,375,857	87.03	10.69	0.75
4_2_1R	41,035,914	35,495,851	86.5	11.3	0.76
4_2_Non	41,939,844	36,891,547	87.96	10.71	0.65

#### 2.9.3 Bioinformatics analysis

Quality control was assessed using Fastqc (v0.11.5). Reads were trimmed for adapters, low quality 3` and a minimum length of 20 using CUTADAPT (v1.12). 100 bp single reads were aligned to the Mus Musculus (GRCm38) reference genome (http://ftp.ensembl.org/pub/release94/fasta/mus_musculus/dna/Mus_musculus.GRCm38.dna.toplevel.fa.gz) and annotation file (http://ftp.ensembl.org/pub/release-101/gtf/mus_musculus/Mus_musculus.GRCm38.101.gtf.gz) using STAR (v2.6.0). The number of reads per gene was counted using Htseq-count (v0.11.2) with “reverse” mode. Normalization and differential expression analyses were conducted using DESeq2 R package (v1.34.0). The threshold for significantly differentially expressed genes was determined based on an adjusted p-value ≤ 0.05 and the “base-mean independent filtering” threshold, which is calculated by the DESeq2 algorithm for each comparison. FDR was calculated using the default approach of DESeq2, Benjamini-Hochberg.

#### 2.9.4 Interactions, pathways and networks

The DEG lists were imported and analyzed using Ingenuity Pathway Analysis (IPA) software (Qiagen), for pathways, networks, etc.

### 2.10 Statistical analysis

Statistical analyses were performed using Microsoft Excel. Values reported in figures are expressed as the standard error of the mean, unless otherwise indicated. For normally distributed datasets observed between groups, we used 2-tailed Student’s t-test. *p*-values < 0.05 were considered significant. * *p*-values < 0.05, ** *p*-values < 0.01.

## 3 Results

### 3.1 IGF1R signaling in myeloid-differentiated DC cells

The role of the IGF1 signaling pathway in DC maturation remains unclear. We previously reported that inhibition of IGF1R signaling in monocytes may lead to enhanced DC differentiation (40, 41). Thus, in the current study we initially checked whether the IGF1R pathway could regulate DC maturation.

#### 3.1.1 Effect of IGF1R inhibition on *in-vitro* DC differentiation

**Figure 1:**
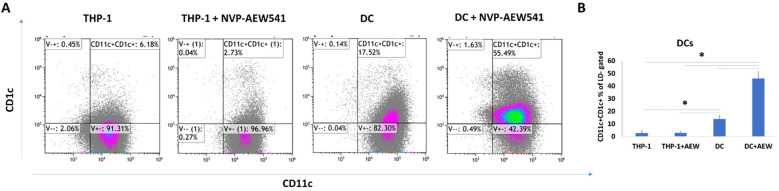
AEW increased DC differentiation. DC differentiation was measured using FCM assays. Human leukemic THP-1 cells were differentiated to DC by treatment with 40 ng/ml IL-4, 20 ng/ml GM-CSF, 20 ng/ml TNFα and 200 ng/ml ionomycin for 48 h, and with 5 μM of AEW for 48 h. THP-1, AEW-treated-THP-1, DC and AEW-treated-DC were stained with CD11c+CD1c+ DC markers. A representative experiment is presented **(A)** CD11c+CD1c+ DC induced rate in THP-1, AEW-treated-THP-1, DC and AEW-treated-DC after 24 h. **(B)** statistical analysis of the abundance of CD11c+CD1c+. The graph represents the average CD11c+CD1c+ frequency of three independent experiments. * *p <*0.05. Bars represent SEM values.

**Figure 2:**
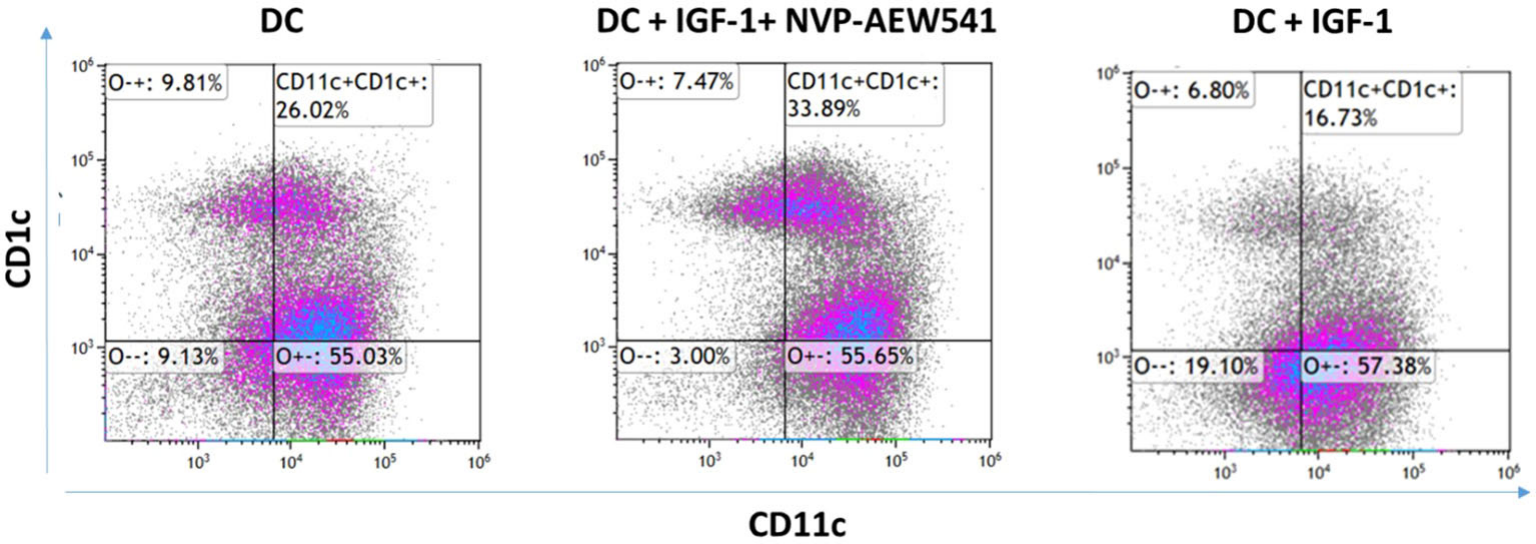
IGF1 treatment decreased differentiation of DC. Human THP-1 cells were differentiated to DC by treatment with 40 ng/ml IL-4, 20 ng/ml GM-CSF, 20 ng/ml TNFα and 200 ng/ml ionomycin for 48 h. The THP-1 cells were treated with 50 ng/ml IGF-1 for 10 min and with 5 μM of AEW for 48 h. DC were stained with CD11c+CD1c+ markers. A representative experiment is presented. The abundance of DCs without treatment (left), DC abundance with IGF-1 and AEW treatments (middle), and DC abundance with IGF-1 treatment (right). The experiment was repeated 3 times.

To investigate the involvement of IGF1R in DC differentiation, THP-1 cells were differentiated into DC in the presence or absence of 5 μM AEW. Undifferentiated THP-1 and AEW-treated-THP-1 were used as additional controls. After a 24 h differentiation protocol, the cells were stained for human DC markers CD11c and CD1c and analyzed by flow cytometry (FCM) assays. As shown representatively in **Figure 1A**, we found a significant increase in the frequency of DCs upon AEW treatment compared to untreated DCs. Our results show that DC frequency increased by 43%, 40% and 32% in DC pretreated with AEW compared to untreated DC, THP-1 and AEW-treated-THP-1, respectively (**Figure 1B**). To further confirm these results, DC differentiation under IGF1 treatment was measured and analyzed using FCM assays. Notably, the frequency of CD11c+CD1c+ DC differentiation decreased following IGF1 treatment (**Figure 2**, right panel) compared to control DC (**Figure 2**, left panel). In contrast, when differentiated DC were exposed to AEW directly after IGF1 treatment, we found that the prevalence of CD11c +CD1c+ increased (**Figure 2**, middle panel) compared to control DC and IGF1-treated-DC. These results suggest that the inhibitor can overcome the negative effect of IGF1 on differentiation.

#### 3.1.2 Differentiated AEW-treated DC reduce ovarian cancer cell proliferation

**Figure 3:**
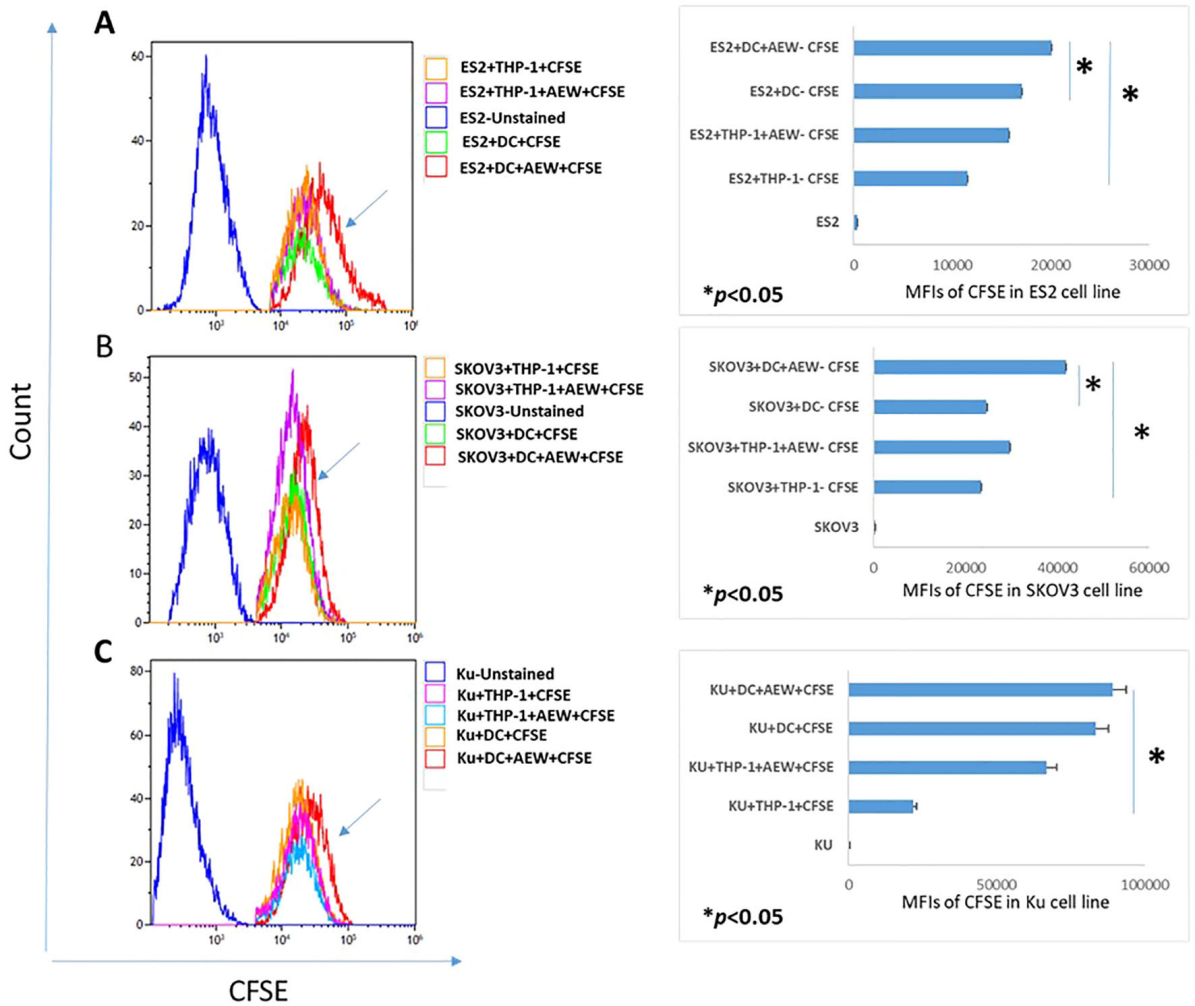
DC treated with IGF1R inhibitors affect OC cell line proliferation. Human leukemic THP-1 cells were differentiated to DC by adding 40 ng/ml IL-4, 20 ng/ml GM-CSF, 20 ng/ml TNFα and 200 ng/ml ionomycin for 48 h and 5 μM of AEW for 48 h. Differentiated DC, IGF1R-treated-DC, THP-1 or IGF1R-treated-THP-1 were each co-cultured with CFSE-pre-labeled OC cell lines for 48 h. Representative flow cytometry histograms of CFSE proliferation assays for **(A)** ES2 cells (left) and flow cytometry analysis for ES2 cells (right). **(B)** CFSE dilution assay for SKOV3 cell proliferation (left) and flow cytometry analysis for SKOV3 cells (right). **(C)** CFSE dilution assay for Ku cell proliferation (left) and flow cytometric analysis for Ku cell (right). The graphs represent OC cell lines CFSE MFI from three independent experiments. * p<0.05. Bars represent SEM values.

Next, we examined whether the enhanced DC differentiation under IGF1R signaling inhibition affected the growth of OC cells. The effect of differentiated AEW-treated DC on EOC cell growth was evaluated using a CFSE proliferation assay. CFSE labeled ES2, SKOV3 and Ku cells were co-cultured for 48 h with inhibitor-treated DC and the EOC proliferation rate was examined with FCM assays and compared to the control groups. We found 42%, 21% and 15% decreases in the CFSE mean fluorescence intensity (MFI) of ES2 cells co-cultured with THP-1, THP-1+AEW and with DCs, respectively as compared to ES2 co-cultured AEW-treated-DC cells (**Figure 3A**). Similarly, we found that SKOV3 cells decreased the CFSE signal by 44.3% when co-cultured with THP-1, by 29.3% with THP-1+AEW and by 41.6% with DC compared to co-culture of AEW-treated DC (**Figure 3B**). In co-cultured Ku cells, we found a decrease in MFI in the THP-1 and DC co-culturing compared to AEW-treated-DC co-culture, but the differences were not significant (**Figure 3C**). Collectively, both ES2 and SKOV3 co-cultured with DC pretreated with AEW showed less proliferation, as inferred from their relative CFSE signal. Therefore, our data indicate that inhibiting IGF1R signaling increases differentiation into DC, which leads to decreased growth of OC cells.

### 3.2 The combined effect of IGF1R and PD-1 inhibitors on EOC proliferation and TME

In various tumors, including OC, DC comprise up to 40% of the infiltrating immune cells (42). Nevertheless, knowledge concerning their role in the TME is sparse. Our *in-vitro* studies suggest the possible involvement of IGF1R signaling in restraining DC maturation, which consequently prevents an immune response. The impact of IGF1R-targeted therapy on OC has been widely investigated, but applying IGF1R targeting in clinical studies failed to show significant benefit. Immune checkpoint inhibitors are currently being targeted in a variety of human malignancies and one of the promising immune checkpoint pathways targeted clinically is PD-1. However, in OC, blocking PD-1 signaling was found to be less successful. Combining IGF1R-targeted therapy with immunotherapy to treat OC has a strong therapeutic potential. Of note, the *in-vitro* approach lacks a full active immune system and therefore, animal studies should be implemented to examine the effectiveness of the combined treatment.

#### 3.2.1 Combination therapy additive effect has positive outcomes against EOC

**Figure 4:**
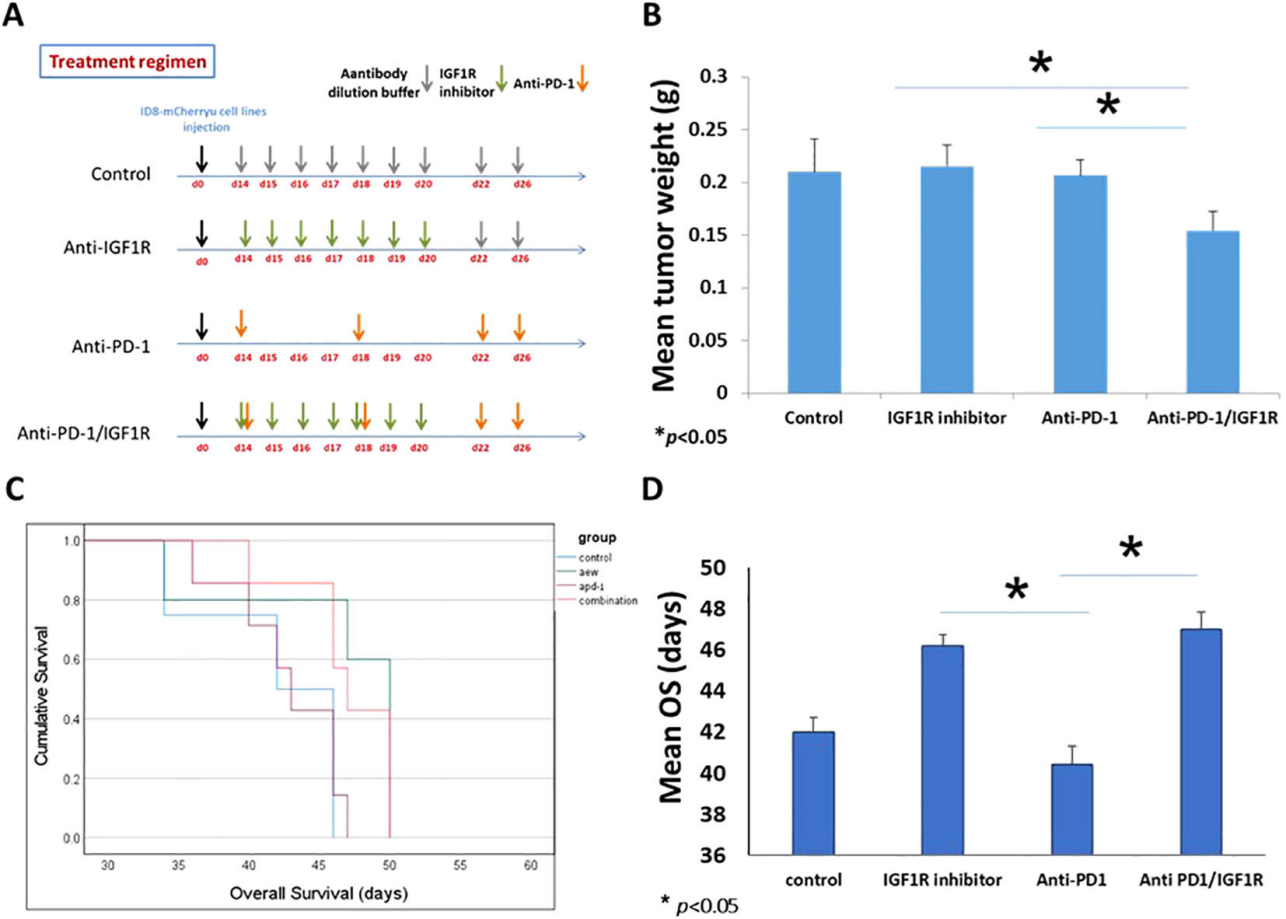
Treatment regimen and the presentation of ID8 ovarian cancer. 32 C57BL/6 mice were inoculated intraperitoneally with 3.5 *10^6^ ID8-mCherry cells, and randomly divided into 4 groups. 14 days after tumor cell injection, mice were treated with either dilution buffer as control, 50 mg/kg IGF1R inhibitor daily for a week, 200 µg anti-PD-1 twice per week for 2 weeks, or anti-PD-1/IGF1R. **(A)** Schematic regimen for single and combined treatment. **(B)** Graph representing the mean tumor weight in the single treatments and the combined treatment. **(C)** The survival of tumor-bearing mice was monitored by Kaplan-Meier analysis and statistical analyses were performed with Log rank test. **(D)** Graph of the mean survival time. Bars represent mean ± SEM.* *p* < 0.05.

To test our hypothesis that IGF1R blocking and anti-PD-1 therapies might work in synergy, and to explore the effect of the combined treatment on the TME of EOC, 32 C57BL/6 female mice, 8 weeks old were injected IP with 3.5*10^6^ ID8-mCherry. This established EOC mice model has clinical relevance, as ascites formation and metastases in the peritoneal cavity were reported previously (42). Two weeks post-tumor injection, mice were randomized into 4 groups (8 mice per group): untreated, treated with anti-PD-1, treated with IGF1R inhibitor (AEW), and treated with a combination of anti-PD-1 and the IGF1R inhibitor (anti-PD-1/IGF1R). PD-1 inhibitor was administered IP at a dose of 200 μg twice per week for 2 weeks (43–45). IGF1R inhibitor was administered by oral gavage, at a dose of 50 mg/kg, twice daily for 7 days (46, 47). Untreated control mice were injected with an antibody dilution buffer (**Figure 4A**). All inoculated mice were followed weekly for ascites and when signs of distress appeared, the mice were sacrificed, and the tumor was retrieved and analyzed. Altogether, 23 of the 32 mice developed a tumor and ascites: 4 in the control group, 5 in the IGF1R inhibitor group, and 7 each in the anti-PD-1 and combined groups. Analysis of the tumors revealed that the combined anti-PD-1/IGF1R treatment led to a significant decrease in the mean tumor mass compared to mice treated with either the anti-PD-1 or the IGF1R inhibitor (34% and 40% decreases, respectively; **Figure 4B**). Intriguingly, there was no significant decrease in mean tumor weight in the combined therapy (0.209g) compared to control group (0.153g). It is important to realize that the control group mice had developed ascites and were sacrificed two weeks before the animals in the IGF1R inhibitor group and the combined group. Consequently, the tumor weights were not measured at the same time, which might explain why the change in tumor weights in the IGF1R inhibitor group and combined group were not significantly different compared to the control. Further, this early ascites development strengthens our hypothesis that the combined treatment may improve survival in EOC mice. In addition, we followed ascites development and OS and found that the control mice and anti-PD1-treated mice developed ascites similarly 35 days after tumor cell inoculation. Notably, in the mice treated with IGF1R inhibitor, ascites development was relatively delayed to 45 days after tumor cell inoculation. The combined anti-PD-1/IGF1R treated group showed no further inhibition of ascites development and behaved similarly to the IGF1R inhibitor-treated group. Interestingly, Kaplan-Meier survival curve showed that the OS rate of the anti-PD-1/IGF1R treated mice was 11% better compared to the controls and 15% better than the anti-PD1 treatment. In contrast, there was no further improvement in the OS of mice receiving the combined treatment compared to the IGF1R inhibitor treatment (**Figures 4C, D**). Altogether, the combined treatment resulted in a decrease in the mean tumor mass compared to mice treated with either the anti-PD-1 or the IGF1R inhibitor, and delayed ascites development compared to the control and the anti-PD-1, and improved survival in the combined treatment compared to anti-PD-1 treatment alone.

#### 3.2.2 Combined treatment induced immune cell infiltration in TME

**Figure 5:**
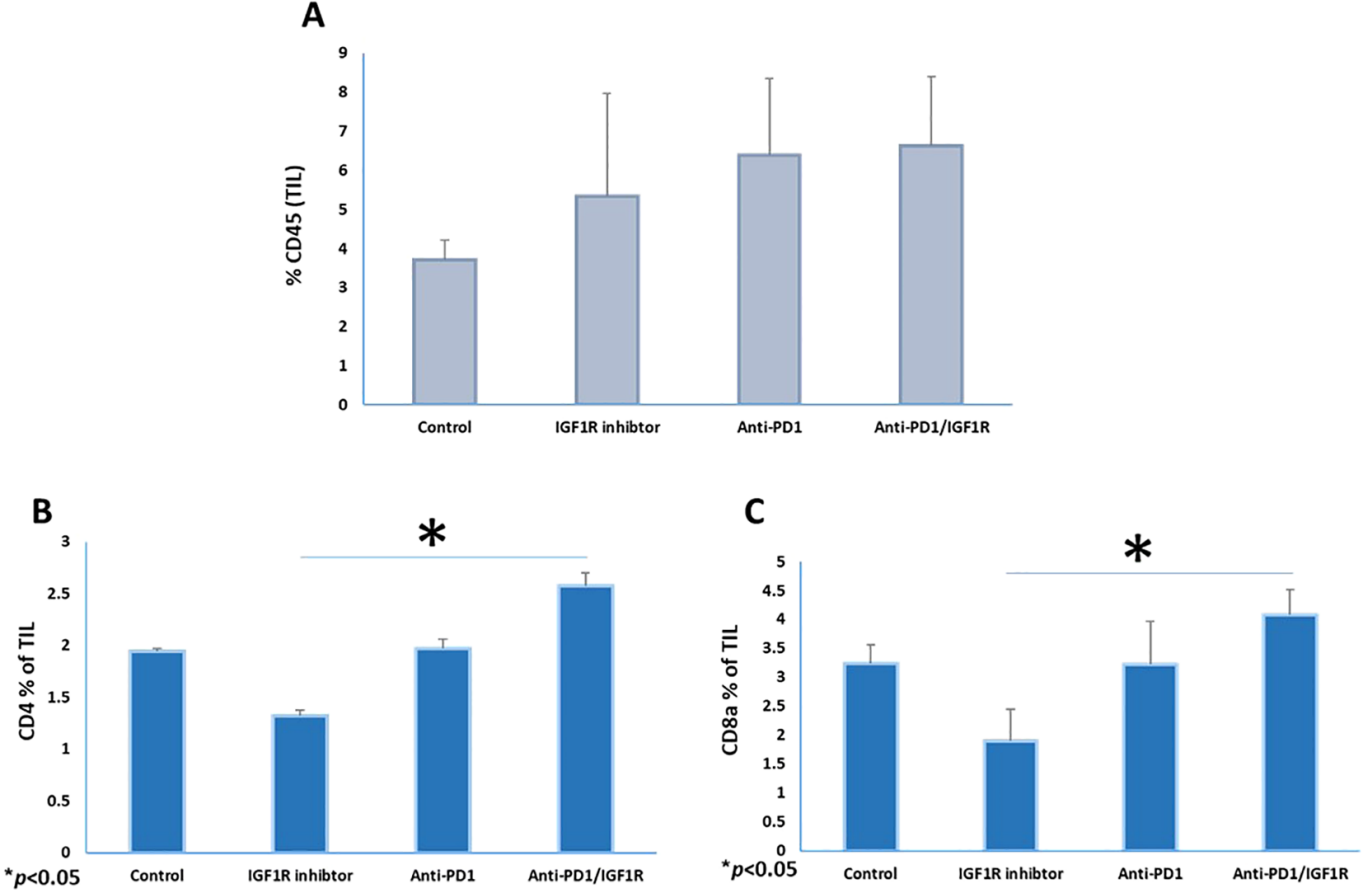
Co-inhibition of PD-1 and IGF1R increased CD8 T-cell populations in the ovarian cancer microenvironment. Three tumors from each group were resected and stained with CD45+, CD4+ and CD8a+ markers. The mice were treated with either dilution buffer as control, 50 mg/kg IGF1R inhibitor daily for a week, 200 µg anti-PD-1 twice per week for 2 weeks, or anti-PD-1/IGF1R. The prevalence of **(A)** leukocyte (CD45+), **(B)** T-helper (CD4+) and **(C)** T-cytotoxic (CD8a+) in single cell suspensions from ID8 tumor-bearing mice are shown. Data were obtained from FCM assays, with 3 mice/group. All cell subtypes were CD45+ gated. Bars represent mean ± SEM.* *p* < 0.05.

Next, we tested whether the combined inhibition of PD-1/IGF1R was associated with reversal of immune response suppression and enhanced TIL. For that purpose, the frequencies of leukocytes (CD45), T cells (CD8a and CD4) and DC (CD86, CD11c, CD8a and CD11b) of 3 tumors from each group were measured and analyzed using FCM assays. Two weeks after treatment completion, peritoneal tumors were resected, and single cell suspensions were prepared. The abundance of the CD45+ TIL in the anti-PD-1/IGF1R combined treatment group was the same as in both single treatment groups (**Figure 5A**). Interestingly, the amounts of CD4 and CD8a T cells were increased by 82% and 87%, respectively, following administration of anti-PD-1/IGF1R, compared to the IGF1R blocking single treatment (**Figures 5B, C**).

#### 3.2.3 DC are present in the microenvironment of an EOC mice model

**Figure 6:**
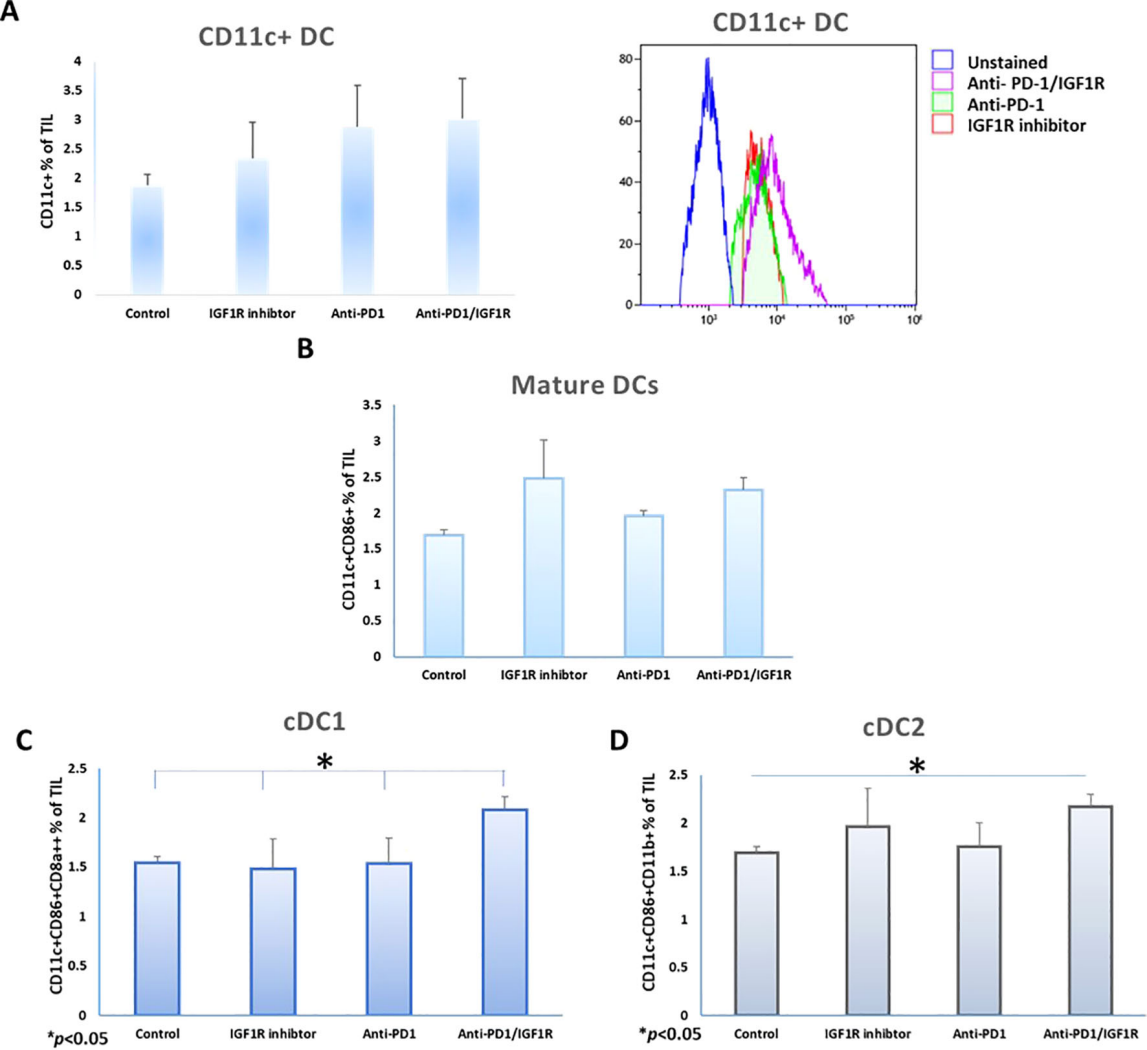
Co-inhibition of PD-1 and IGF1R increase DC prevalence in the ovarian cancer microenvironment. Mice were inoculated intraperitoneally with 3.5*106 ID8 tumor cells, mice were treated with either dilution buffer as control, 50 mg/kg IGF1R inhibitor daily for a week, 200 µg anti-PD-1 twice per week for 2 weeks or anti-PD-1/IGF1R. Three tumors from each group were harvested and stained with CD45+, CD11c+ and CD86+, CD8a+ and CD11b markers. DC (CD45+CD11c+), and classic dendritic cells (cDC1 and cDC2) were quantified in cells isolated from tumors. The rates of **(A)** CD11c+ DC (left) and histogram of CD11c DC expression on CD45+ cells (right), **(B)** CD86 positive CD11c DCs, **(C)** cDC1 (CD11c+CD86+CD8a+CD11b-) and **(D)** cDC2 (CD11c+CD86+CD11b+) isolated from ID8 tumor-bearing mice, were measured and analyzed using FCM assays, 3 mice/group. All cell subtypes were gated on CD45+. Bars represent mean ± SEM. *p < 0.05.

We examined the frequencies of total CD11c+ DC and conventional dendritic cell (cDC) subtypes following the treatments. As shown in **Figure 6A**, there was no change in the abundance of the CD11c+ DC population following the combined treatment, compared to each of the single treatments. In addition, after the combination therapy, there was no significant change in the expression of the DC maturation marker CD86 on gated CD45+CD11c+, compared to each single treatment (**Figure 6B**). Next, we investigated the infiltration of cDC in the TME. In mice, the cDC are divided into two major subsets: cDC1 and cDC2, based on the expression of nuclear transcription factors and their surface markers. cDC1 perform cross-presentation of antigens to MHC class I and activate the immune responses of T cytotoxic cells, while the cDC2 activate CD4+ T cells and mediate either Th2- or Th17- immune responses (48). Examining these subsets in the treated groups showed that the abundance of the cDC1 subset, defined as CD45+CD11c+CD86+CD8a+, was significantly increased by 35%, 64% and 35%, respectively, following the combined treatment, as compared to control, IGF1R inhibitor and anti-PD-1 treatments (**Figure 6C**). The cDC2 subset, defined as CD45+CD11c+CD86+CD11b+, increased significantly by 29% following the combined treatment compared to the control. However, there was no significant change compared to IGF1R inhibitor (13%) and anti-PD-1 (24%) treatments (**Figure 6D**).

In conclusion, the combined treatment of anti-PD-1 and IGF1R blockade led to an increase in the abundance of EOC cDC subtypes. The increase in cDC might be biologically functional, as we found that there was an increase in TIL under the same treatment. Overall, the combined treatment showed improved potential to reactivate the immune response in our EOC model.

#### 3.2.4 Determining the efficacy of anti-PD-1/IGF1R combined treatment in EOC through gene expression analysis

**Figure 7:**
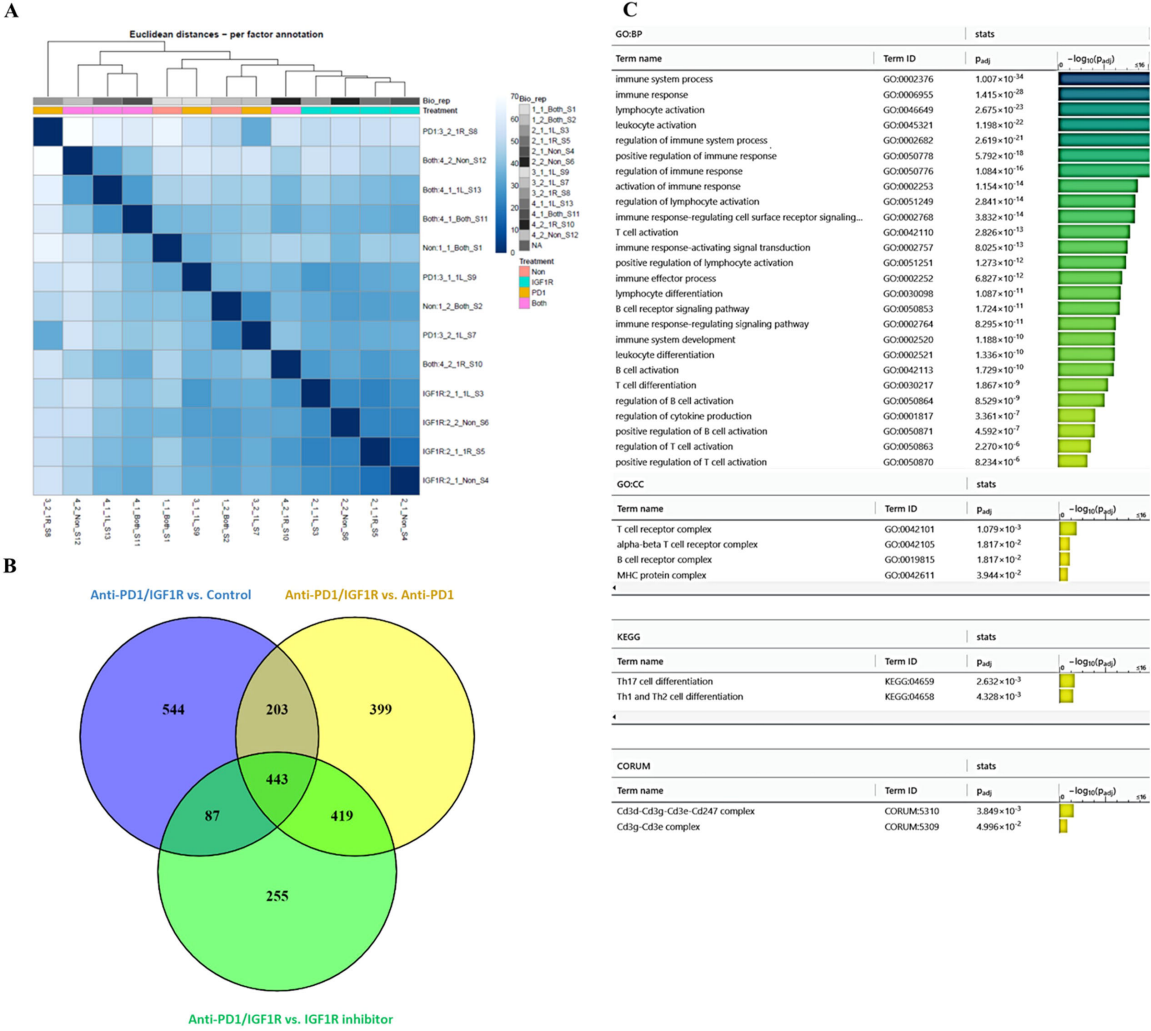
Exploring the effect of combined anti-PD-1/IGF1R treatment on the tumor microenvironment, through RNA-seq. Mice were inoculated intraperitoneally with 3.5*106 ID8 ovarian tumor cells. Tumors were harvested from 2 mice in the control group, 4 mice in the IGF1R inhibitor-treated group, 3 mice in the anti-PD-1-treated group, and 4 mice in the anti-PD-1/IGF1R-treated group. Subsequently, RNA-seq was performed using RNA extracted from the 13 tumors. **(A)** A hierarchical clustering heatmap of the 4 experimental groups based on gene expression RNA-seq. **(B)** A Venn diagram showing the common and unique differentially expressed genes in the combined anti-PD-1/IGF1R treatment group compared to control, IGF1R inhibitor and ant-PD-1 treatments. Target genes were filtered by a fold change >1.5 or < -1.5. **(C)** GO analysis bar graph of the 443 shared genes, showing the enriched biological processes.

**Figure 8:**
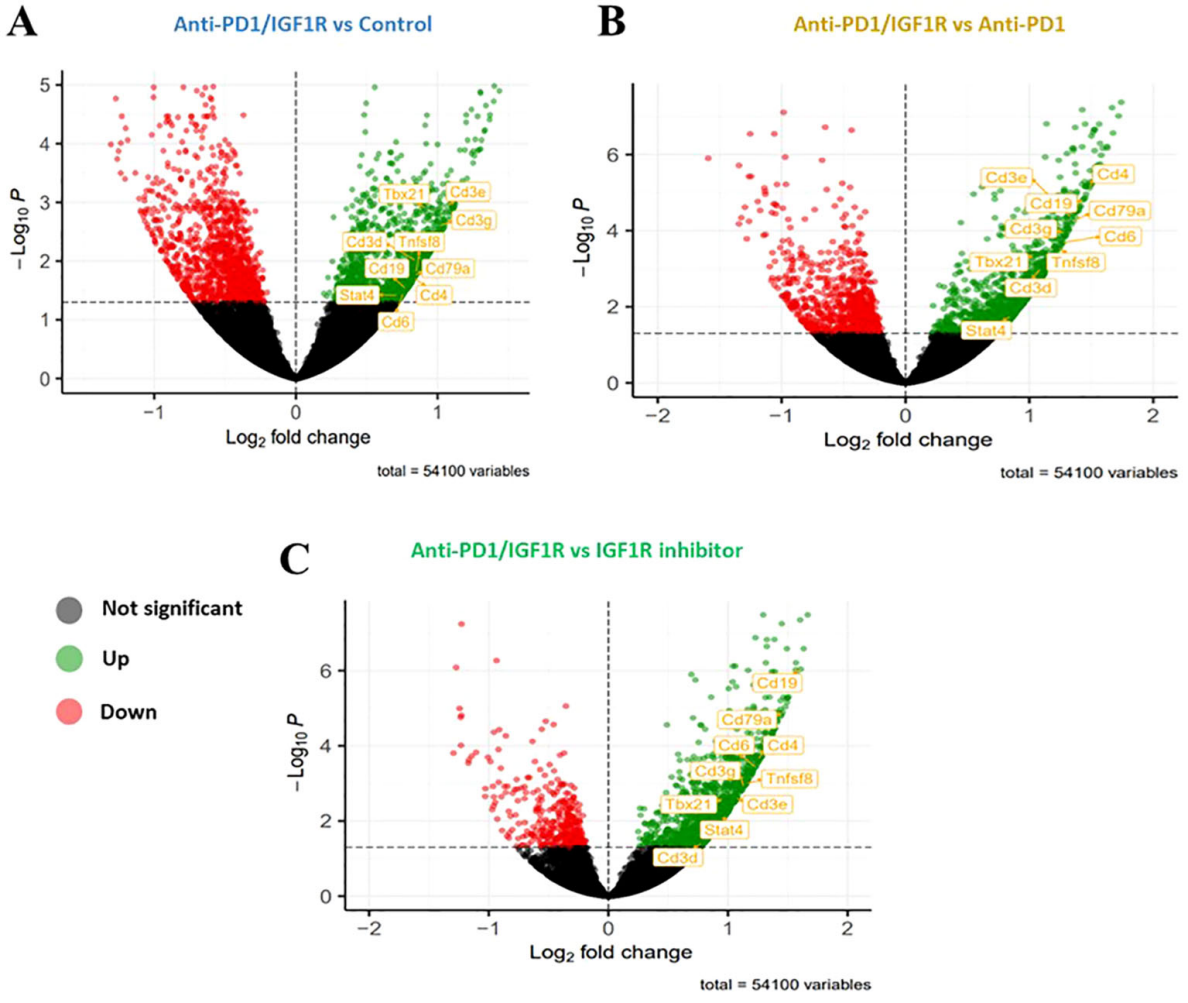
Up- or down-regulated target genes significantly affected by anti-PD1/IGF1R treatment. Mice were inoculated intraperitoneally with 3.5*106 ID8 ovarian tumor cells (n=8/group). Tumors were harvested from 2 mice in the control group, 4 mice in the IGF1R inhibitor-treated group, 3 mice in the anti-PD-1-treated group, and 4 mice in the anti-PD-1/IGF1R-treated group. Subsequently, RNA-seq was performed using RNA extracted from the 13 tumors. Volcano plots showing expression fold changes (X-axis) versus *p* value (Y-axis). Significantly up-regulated genes are presented in green on the volcano’s right side and the significantly down-regulated genes are presented in red on the left side of the volcano. **(A)** anti-PD-1/IGF1R treatment compared to Control. **(B)** anti-PD-1/IGF1R treatment compared to Anti-PD-1 treatment. **(C)** anti-PD-1/IGF1R treatment compared to IGF1R inhibitor treatment.

To identify the battery of genes responsive to the different treatments, RNA-seq was performed using RNA extracted from 13 tumor samples representing the 4 study groups: 2 control tumor samples, 4 IGF1R inhibitor-treated tumor samples, 3 anti-PD-1-treated tumor samples and 4 anti-PD-1/IGF1R-treated tumor samples. The first step in analyzing the sequencing data was to produce a hierarchical clustering heatmap. According to the heatmap shown in **Figure 7A**, the control and anti-PD-1 treatment samples were more alike, while the cluster of IGF1R inhibitor-treated samples was next. Importantly, the combined treatment samples were very different since they were most distantly clustered (**Figure 7A**). Next, we examined the extent of the differences in gene expression by comparing the treatment groups. The anti-PD-1 and IGF1R blocking treatments had modest effects on total gene expression relative to control (1.77% and 3%, respectively). In contrast, the combined anti-PD-1/IGF1R treatment affected total gene expression dramatically: 14.7% relative to the control. Similarly, the combined treatment affected total gene expression by 9.2% and 12.8% relative to the single treatment of IGF1R or PD-1, respectively. Furthermore, we then distinguished between up-regulated or down-regulated target genes. While the combination treatment led to a similar number of up-regulated genes compared to control, IGF1R inhibitor treatments, and PD-1 treatment (7.6%, 7.1%, and 8%, respectively), we found that the ratio between up- and down-regulated target genes was different. In the combined treatment vs. control, 51% of the Differential expression (DE) genes were up-regulated and 49% were down-regulated. This similarity changed to 63% up- and 37% down-regulated genes when comparing combined treatment to the single anti-PD-1 and changed even further to 78% up- and 22% down-regulated genes when comparing combined treatment to the single IGF1R inhibitor treatment (**Table 2**). Next, we wanted to understand the biological meaning behind the list of DE genes and especially those related to the combined treatment. For that purpose, we applied an adjusted fold change cutoff >1.5 and < -1.5 in combined treatment versus the individual treatments and intersected them. The Venn diagram, representing the intersection, identified 443 common genes (**Figure 7B**). Out of these 443 genes 414 were up-regulated while only 29 were down-regulated. Next, we performed a Gene Ontology (GO) analysis on these 443 genes to look for enriched biological processes. Interestingly, the most significant differential biological process terms derived from the up-regulated genes were immune response, leukocyte activation in general and, particularly, lymphocyte activation in B cells, T cells, Th1 and Th17 (**Figure 7C**). Among the 29 down-regulated genes we found no GO enrichment presumably because of a small number of genes. Nevertheless, genes such as Nell2, Smarca1, Slc6a14 and Grhl3 which were highly downregulated by the combined treatment were reported to contribute to tumor progression. Furthermore, the volcano plots demonstrated significantly increased expression of the CD4, CD6, CD3e, CD3d, CD3e, CD19, CD79a, TBx21 and Stat4 genes in the combined treatment compared to each individual treatment. These genes, encode surface proteins of T and B cells, as well as NK cells, or cytokines, which play a critical role in immunity (**Figure 8**).

**Table 2:** Summary of pair-wise differential expression analysis.

Comparison	Total Genes^a^	Tested^b^	Significant Up	Significant Down	Total DE Genes
IGF1R inhibitor vs. Control	54100	15406	199	276	475
Anti-PD-1 vs. Control	54100	15963	118	165	283
Anti-PD-1/IGF1R vs. Control	54100	17489	1325	1253	2578
Anti-PD-1/IGF1R vs. IGF1R inhibitor	54100	17718	1263	361	1624
Anti-PD-1/IGF1R vs. Anti-PD-1	54100	17844	1430	857	2287

Differential expression (DE) analysis: ^a^Total number of genes: all the genes present in the annotation file; ^b^Tested: the number of genes that entered the statistical analysis and received a final adjusted p value.

## 4 Discussion

Ovarian cancer is a lethal malignancy that can suppress anti-tumor immune responses by inducing severe TIL dysfunction, including DC dysfunction in the host (6). TIL are thought to play an important role in the control of tumor growth by activating an anti-tumor immune response (15, 49, 50). TIL consist predominantly of CD8+ T cells, CD4+ T cells, DC, macrophages and regulatory T cells (51). DC are uniquely able to activate T cytotoxic activity (6). However, in tumorigenesis, tumor cells can release factors that inhibit DC maturation and function, which lead to lack of activation of prime T cells (52). Several studies reported a correlation between the presence of intratumoral T cells and improved clinical outcomes in advanced OC (50, 53). Analysis of 186 samples of advanced stage OC revealed a five-year survival rate of 38% in patients with detectable intraepithelial CD3C TIL compared to 4.5% in patients with no TIL (50). Another study found that the presence of intraepithelial CD8+ TIL improved survival in OC patients (53). To date, multiple mechanisms responsible for DC dysfunction in OC have been identified and characterized to elicit therapeutic immunity and control OC progression (54). Studies showed that the IGF axis regulates DC maturation and suppresses DC-mediated immunity in OC (26, 55). However, the involvement of IGF1R signaling in tumor-infiltrating immune cells at the TME is still not clear. IGF1 signaling has been demonstrated to play a crucial role in the development and progression of cancers, including ovarian (56). As a corollary, clinics had high expectations regarding IGF1R targeting strategies. Unfortunately, these hopes were tempered by a lack of significant efficacy in clinical studies.

Our previous studies provided evidence that inhibition of the IGF1R signaling pathway in monocytic cell lines (THP-1 and HL-60) reduced EOC cell migration (40, 41). We demonstrated that IGF1R inhibitor led to a marked decrease in ES2 and SKOV3 cell migration when co-cultured with DC pretreated with IGF1R inhibitor, as compared to treated THP-1, untreated THP-1 and untreated DC. Based on our data, we postulated that targeting the IGF1 axis in monocyte cells would increase DC maturation. This concept placed the IGF1R-targeted therapy in a new perspective. In the current study, we showed that IGF1R inhibition in THP-1 cells induced DC differentiation, whereas adding IGF1 prevented it (**Figures 1**, **2**). Hence, the direct effect of DC on cancer cells may be influenced by the IGF1 axis activation status. We also showed that co-culture of EOC cells with DC pretreated with IGF1R inhibitor reduced EOC proliferation (**Figure 3**). These findings support those of a previous report showing that DC derived from human PBMCs can directly inhibit the proliferation of various human tumor lines and can also induce an antitumor effect by stimulating T lymphocytes (57). Interestingly, Wu et al. reported that IGF1R phosphorylation was correlated with poor immunosurveillance, as indicated by low infiltration of CD8+ T cells and high frequency of regulatory T cells in patients with breast cancer (58). Furthermore, Xuan et al. showed that exposure of DC to IGF1 was followed by decreased transcription and expression of the anti-aging hormone klotho (55). Klotho modulates the influx of Ca^2+^, which plays a key role in maturation and apoptosis in immune cells, including DC (59). Further research is needed to understand the role of IGF1 signaling pathway involvement in the immune system.

In line with the developing immunotherapeutic approaches, we suggested that co-inhibition of IGF1R and PD-1 may reverse immune escape in EOC patients and could improve response to these targeted therapies. Interestingly, the fact that the IGF1R and the PD-1 share the PI3K/AKT as a downstream signaling pathway reinforces this perception. Of note, the PI3K/AKT signaling pathway is frequently dysregulated in tumors and has now become an important target for anticancer treatment (60, 61). Moreover, cell proliferation, survival, and invasion are all regulated by PI3K/AKT.

The results of our murine study showed that the combination anti-PD1/IGF1R treatment significantly reduced the tumor burden by 40% compared to IGF1R inhibitor and 34% with anti-PD-1 treatments (**Figure 4B**) and achieved a higher survival rate compared to the anti-PD1 treatment alone (**Figures 4C, D**). Despite the significantly decreased tumor weight observed in combined group compared to IGF1R inhibitor group, the OS rate appears similar. This can be explained by the development of ascites or additional parameters that can affect OS rate. Nonetheless, these results clearly show an additive antitumor effect of the combined therapy in an EOC mouse model and suggest a novel approach for individualized treatment for EOC patients. A previous *in-vivo* study showed that anti-PD-1 antibodies alone reduced tumor burden and improved T cell function, but when the PD-1 receptor on the DC was blocked, more immune regulatory cytokines were released (62). However, Wei et al. reported a lack of T cell response when using a PD-1 inhibitor alone (63). In line with our results, Ajona et al. showed that the combined IGF1R and PD-1 inhibition synergistically reduced tumor growth in a lung cancer mouse model (64). Moreover, Wu et al. showed that combining the IGF1R inhibitor picropodophyllin-PPP with PD-1 blockade enhanced the efficacy of anticancer chemotherapies in a breast cancer mouse model (58).

Recent preclinical studies showed the promising therapeutic potential of reversing the immune response by activating the T cells, B cells, DC and NK cell in the TME, when anti-PD-1 is combined with other checkpoint inhibitors, immunostimulatory molecules, or cytotoxic agents in EOC models (44–46, 65). Krempski et al. suggested that in advanced disease, PD-1 expression increased in DC. They also showed that CD11c+ DC are the predominant suppressor cells in the TME (62). The conventional DC, cDC1 and cDC2, play critical roles in promoting antitumor effects by presenting tumor antigens (66). However, tumor cells can escape from the immune response by suppressing the anti-tumor immunity and provoking DC dysfunction in the TME (67). Another study showed that DC in the ovarian TME play a role in inducing immune suppression and promoting tumor formation. This immune suppression is mediated by factors such as IL-8, TNF-α, and IDO, which are produced by the dysfunctional DC (68). These studies suggest that the number of efficient mature DC and tumor-specific T cells should be increased, to shift the balance from immunosuppression towards immune surveillance.

Analysis of the cellular components of the TME in the current study showed that a single anti-IGF1R treatment lowered the number of CD8+ and CD4+ T cells. Anti-PD1 treatment alone had no effect on the number of CD8+ and CD4+ T cells, whereas combination treatment significantly increased the number of CD8+ and CD4+ T cells compared to control (**Figures 5B, C**). Flow cytometry analysis revealed a higher expression rate of CD11c+ CD86+ CD11b+ (cDC1) in anti-PD1/IGF1R treatment compared to single treatments, and the single treatment groups showed similar expression rates compared to control (**Figure 6C**). Previous studies have demonstrated the crucial role of DCs in recruiting effector CD8+ T cells within the TME (69, 70). Considering the relatively low prevalence of immune cells in the TME, even a minor increase in DC percentage could potentially trigger an anti-tumor response. Hence, our results strengthen the evidence supporting the additive effect of the combined anti-PD-1/IGF1R treatment. Further, we propose that conducting experiments involving a decrease in DC would reveal the direct impact of DCs on the TME. This decrease is expected to result in reduced antigen presentation, hindering the proper activation of T cells. This, in turn, can lead to the dominance of immunosuppressive factors, allowing the tumor to evade immune surveillance and persistently grow. All these effects collectively contribute to a diminished effective response against the tumor.

RNA-seq analysis showed that the anti-PD-1/IGF1R treatment is characterized by an induced immune response, as compared to control, IGF1R inhibitor and anti-PD-1 treatments (**Figure 7**). In contrast, this increase in immune system genes was not observed with IGF1R inhibitor and anti-PD-1 treatments compared to control (**Figure 8**). We found that treating ovarian tumor-bearing mice with a combination of anti-PD-1/IGF1R led to a more potent response, as reflected by higher rates of up-regulated and down-regulated gene expression, compared to separate treatments (**Figure 7B**). Moreover, GO analysis indicated that the combined treatment enhanced the anti-tumor immune response by increasing the amounts of T cells, B cells and activated NK cells (**Figures 7C**, **8**). A possible limitation of this study includes a relatively small sample size which may not provide sufficient power for the gene expression analysis.

## 5 Conclusions

Taken together, the results presented here provide evidence of a novel function of IGF1R and PD‐1 in regulating the immune response of EOC. We demonstrated that IGF1R inhibition elicits a potent antitumor immunity and that the combination with PD-1 blockade leads to tumor regression in EOC mice. Moreover, we showed that the combined regimen achieved stronger immune responses in an EOC mouse model as compared to monotherapy. To our knowledge, this is the first study to investigate the additive effect of PD-1 and IGF1R inhibition in EOC. We also developed new tools to study the role of IGF1R in EOC. Our *in-vitro* and *in-vivo* data may serve as starting points for the development of personalized therapy for this devastating disease.

## Data Availability

The analyzed data sets generated during the present study are available from the corresponding author on reasonable request.
